# Associations between child marriage and food insecurity in Zimbabwe: a participatory mixed methods study

**DOI:** 10.1186/s12889-023-17408-7

**Published:** 2024-01-02

**Authors:** Katherine Gambir, Abel Blessing Matsika, Anna Panagiotou, Eleanor Snowden, Clare Lofthouse, Janna Metzler

**Affiliations:** 1 Women’s Refugee Commission, 15 W 37th Street, 9th Floor, New York, NY 10019 USA; 2 Independent Research Consultant, Harare, Zimbabwe; 3 Plas Eirias Business Centre, The Cynefin Co, Abergele Road Colwyn Bay, Conwy, LL29 8BF UK; 4 Plan International, Dukes Court, Block A, Duke Street, Woking, Surrey, GU21 5BH UK; 5 Columbia University Mailman School of Public Health, New York, USA

**Keywords:** Food insecurity, Child marriage, Early marriage, Forced marriage, Child protection, Humanitarian, Adolescents, Nutrition, GBV, Zimbabwe

## Abstract

**Background:**

Child marriage is a global crisis underpinned by gender inequality and discrimination against girls. A small evidence base suggests that food insecurity crises can be both a driver and a consequence of child marriage. However, these linkages are still ambiguous. This paper aims to understand how food insecurity influences child marriage practices in Chiredzi, Zimbabwe.

**Methods:**

Mixed methods, including participant-led storytelling via SenseMaker® and key informant interviews, were employed to examine the relationship between food insecurity and child marriage within a broader context of gender and socio-economic inequality. We explored the extent to which food insecurity elevates adolescent girls’ risk of child marriage; and how food insecurity influences child marriage decision-making among caregivers and adolescents. Key patterns that were generated by SenseMaker participants’ interpretations of their own stories were visually identified in the meta-data, and then further analyzed. Semi-structured guides were used to facilitate key informant interviews. Interviews were audio-recorded, and transcribed and translated to English, then imported into NVivo for coding and thematic analysis.

**Results:**

A total of 1,668 community members participated in SenseMaker data collection, while 22 staff participated in interviews. Overall, we found that food insecurity was a primary concern among community members. Food insecurity was found to be among the contextual factors of deprivation that influenced parents’ and adolescent girls’ decision making around child marriage. Parents often forced their daughters into marriage to relieve the household economic burden. At the same time, adolescents are initiating their own marriages due to limited alternative survival opportunities and within the restraints imposed by food insecurity, poverty, abuse in the home, and parental migration. COVID-19 and climate hazards exacerbated food insecurity and child marriage, while education may act as a modifier that reduces girls’ risk of marriage.

**Conclusions:**

Our exploration of the associations between food insecurity and child marriage suggest that child marriage programming in humanitarian settings should be community-led and gender transformative to address the gender inequality that underpins child marriage and address the needs and priorities of adolescent girls. Further, programming must be responsive to the diverse risks and realities that adolescents face to address the intersecting levels of deprivation and elevate the capacities of adolescent girls, their families, and communities to prevent child marriage in food insecure settings.

**Supplementary Information:**

The online version contains supplementary material available at 10.1186/s12889-023-17408-7.

## Background

Unmet basic needs, such as food, are recognized as a universal risk factor for harmful outcomes for adolescents. Conflict and climate-related disasters are considered significant drivers of food insecurity [1]. Yet, little documented analysis of the linkages between food insecurity and protection risks, including child marriage, in humanitarian settings exists [2]. Food insecurity is defined as “a lack of access to the kinds and amounts of food necessary for each member of a household to lead an active and a healthy lifestyle” [3]. Existing evidence-mostly grey literature- indicates that food insecurity can both facilitate and be a consequence of child marriage [4–11]. Child marriage is widely defined as any formal marriage or informal union where at least one spouse is a child under the age of 18 [12]. Due to gender inequality, adolescent girls are disproportionately affected by child marriage compared to boys [12]. The literature [4–11] argues that food insecurity may lead to child marriage as a coping strategy to allocate limited resources to fewer members of the household and/or ease financial burdens. Although six publications [4–6, 8–10] report data from the African context, no publications cite evidence from Zimbabwe specifically. Further, only one publication [4] argues that when adolescents and families do not have enough food to eat, adolescents themselves may resort to extreme coping mechanisms, such as child marriage, in order to acquire food or other basic necessities. Girls who marry at a young age are more likely to suffer from malnutrition, as are children born to adolescent mothers who married as minors [6, 13].

Due to a complex combination of structural factors, such as a deepening economic depression, and climate change, Chiredzi District (Chiredzi) is one of the most chronically food insecure districts in Zimbabwe. In 2021, Chiredzi was classified at Integrated Food Security Phased Classification (IPC) level 3 “crisis,” with 30% of the population severely affected by food insecurity, including during the 2021 peak lean season [15]. Household economic security worsened due to COVID-19 restrictions that resulted in household incomes being reduced by more than 50% [14].

Over one million young women in Zimbabwe were married as children, equal to 34% of young women aged 20–24 years having been married before age 18, and 5% married before age 15 [16]. In Masvingo Province, where the study was conducted, child marriage rates range between 41% and 50% [16]. A study on child marriage (in which this study is nested) among food insecure communities in Chiredzi, a town in Masvingo Province, found that poverty and unmet basic needs, including household food insecurity, were among the key drivers of child marriage [17]. Evidence from both this study and others globally suggest that food insecurity may drive child marriage because practices, such as bride price (gifts or money from the husband-to-be’s family to the girl’s) or dowry practices (gifts or money from the girl’s family to the husband’s or the couple), can influence parents’ decisions on the timing of child marriage [2, 7, 17]. Coupled with gender inequality and discrimination against girls and women, these factors increase parents’ and caregivers’ likelihood to resort to child marriage for economic benefits or survival. Further, since girls do not benefit economically from bride price or dowry practices, they may initiate their own marriages to access economic security through their husband [17–20].

Given the urgency of the global food crisis triggered by the war in Ukraine and worsening climate change [21, 22] it is critical that development actors, humanitarian practitioners, and policy makers use evidence to inform programs and policy to prevent and respond to child marriage. This paper aims to help fill the evidence gaps on the correlation between food insecurity and child marriage by examining among food insecure communities in Chiredzi: (1) the connections through which food insecurity elevates adolescent girls’ risk of child marriage; and (2) how food insecurity influences child marriage practices.

## Methods

### Analytical framework

**Fig. 1:**
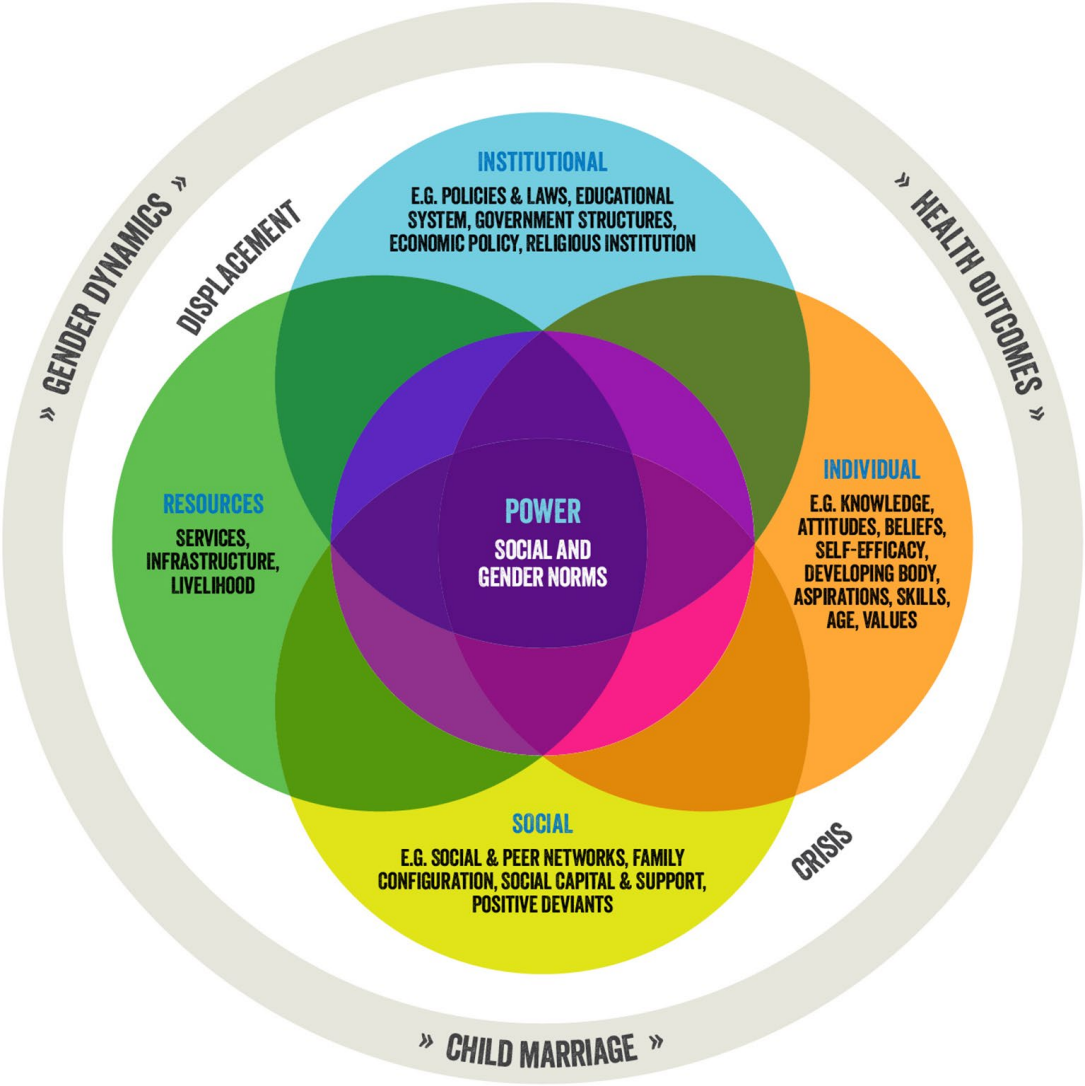
Social norms framework for child marriage in crisis

The research team developed a Social Norms Framework for Child Marriage in Humanitarian Crises (Fig. 1), an adapted analytical framework building on the framework of Pulerwitz et al., 2019 [23]. This Framework explores the critical role of social and gender norms while also recognizing the role of systemic factors in the development and maintenance of dominance, shaping gender and other social structures that affect child marriage practices within a humanitarian crisis. The crisis and displacement level explores how structural and social factors inherent to humanitarian settings interact across all other domains. This framework was used for analytical purposes, but we will highlight the linkages between food insecurity and child marriage using the research questions.

### Exploratory study design

This research was nested within a multi-country study [17], which employed a girl-centered and community-based approach to examine the drivers of child marriage and adaptive capacities of adolescents, their families and communities, and broader systems of support and protection to mitigate the risk of marriage in humanitarian settings. The goal of the study was to use evidence generated to design a contextually relevant program to address child marriage. This paper focuses on food insecurity, which was one of the key themes that emerged from data analysis conducted for the main study. Additional analyses were conducted in this study to explore the connections through which food insecurity elevates adolescent girls’ risk of child marriage and how food insecurity influences child marriage practices. Other interrelated key themes included controlling adolescent girls’ sexuality and decision making, adolescent pregnancy, violence against girls, harmful cultural practices, and COVID-19. This research used mixed methods, including participant-led storytelling via SenseMaker® and key informant interviews (KIIs) to address the research questions. Methods were adapted where necessary to reduce the risk of COVID-19 transmission and in accordance with local government health guidance.

#### SenseMaker

SenseMaker is a mixed methods tool for narrative research. The survey tool was co-developed by an interdisciplinary team. Plan International Zimbabwe staff working in the target communities provided additional feedback to ensure the tool was socially and contextually appropriate. It works by asking participants an open-ended question and recording responses as short, descriptive stories about their lived experiences. The enumerators used the following open-ended story prompt: “Tell me a story about what it’s like for young people to get married in this community.” After the participants shared their story, they interpreted their own story through a series of visual questions to convey deeper layers of meaning to the story. This is called a “signification framework”, which was co-designed with both adolescent girl community members and adults (see Annex 1 for the SenseMaker tool). The signification framework is a community-led approach that facilitates epistemic justice by providing an opportunity for participants to self-signify what is most relevant in their story.

#### KIIs

Semi-structured guides contextualized by Plan Zimbabwe staff and the national research consultant were used to facilitate the KIIs among practitioners delivering or managing adolescent programming. KII guides [Annex 2] aimed to collect data on barriers and facilitators to accessing (and delivering) existing services and programming in target communities, which a focus on child protection and child marriage programming.

### Setting

This study was conducted in Chiredzi, part of Masvingo Province in southwestern Zimbabwe, bordering both South Africa and Mozambique. Chiredzi was selected because (a) it experiences increasingly frequent climate change-related disasters and protracted food insecurity; and (b) it is the location of existing adolescent programming implemented by Plan International (Plan). Around 5.3 million people in Zimbabwe are food insecure as a result of the impacts of climate change and climate-related disasters, and protracted economic instability resulting in a humanitarian crisis. At least 49% of the population is living in extreme poverty [24]. Chiredzi has an arid climate with erratic rainfall, droughts, and high temperatures. Despite these harsh conditions, the primary income-generating activity in Chiredzi is agriculture and livestock farming [25].

#### Data collection

Data were collected between January 2021-April 2021. KIIs were facilitated via the Zoom online communications platform. Plan Zimbabwe staff conducted a service mapping to identify national non-government organizations (NGOs), civil society organizations (CSOs), community-based organizations (CBOs), international non-governmental organizations (INGOs), social service providers, United Nation (UN) actors, and government officials working to advance community and/or adolescent health and protection outcomes in Chiredzi, or other food insecure settings in Zimbabwe. Participants were recruited via maximum variation purposive sampling and invited to participate in KIIs from this mapping until saturation was reached. Individuals were excluded from the study if they worked for Plan. A total of 17 KIIs were conducted with 22 individuals (12 female, 10 male) working for a diverse range of organizations as listed above, with five organizations based in Harare and 12 Chiredzi-based organizations. The research team employed maximum variation sampling for SenseMaker data collection to allow for a range of perspectives and data disaggregation based on target participants. Eligible participants included adolescents aged 10 to 19 years old; parents and guardians of adolescents; community gatekeepers (e.g., traditional and community leaders); and local government officials. Participants were recruited from a list of eligible participants per subgroup identified by Plan International Zimbabwe. Participants were selected from among the general population; participation in Plan International Zimbabwe programming was neither an inclusion nor exclusion criterion. The sampling frame was guided by two primary considerations: (1) selecting an urban area for comparison with a rural setting to explore how different socio-economic contexts and access to social services influence findings; and (2) selecting areas that are accessible to the research team from Chiredzi urban.

The research team facilitated two workshops in Chiredzi urban and Chiredzi peri-urban with married and unmarried adolescent girls, as well as with caregivers and other adult community gatekeepers. Trained facilitators conducted participatory group activities to identify dominant issues facing adolescents in the target communities. These issues were incorporated into the final SenseMaker signification framework. This framework is a set of questions that provides an opportunity for the storyteller to analyze and give meaning to their own story [26]. The signification framework was translated from English to Shona and built into the app-based software. The study was overseen by a WRC researcher based in the United States and an independent research consultant in Zimbabwe who also oversaw two study coordinators in Zimbabwe.

The SenseMaker data collection team comprised of 10 women and 10 men with experience in qualitative and/or quantitative data collection in the community and was trained on addressing sensitive topics among communities in crisis settings. SenseMaker activities were facilitated in Shona or Shangani by a facilitator of the same sex as the participant. SenseMaker data collection was held in a private space inside or just outside the participant’s home. The KII data collection team included the WRC researcher, the research consultant, and the two study managers. The WRC researcher facilitated interviews in English, while the other team members facilitated interviews in both English and Shona. All tools were piloted before being used in the study.

#### Analysis

##### Data analysis

KIIs were audio-recorded, and transcribed and translated to English, then imported into NVivo (Version 12 Plus) for coding and analysis. Researchers reviewed all transcripts and wrote memos on emergent themes, which were used to create a list of inductive codes. These codes were used to create a codebook structured by research objectives and the Social Norms Framework for Child Marriage in Humanitarian Crisis, which was then piloted. The researchers independently coded all transcripts according to the codebook, and met regularly to ensure fidelity of the codebook. Additional thematic summaries were created to explore deviation or discordance in the data based on location or organizational affiliation.

**Fig. 2:**
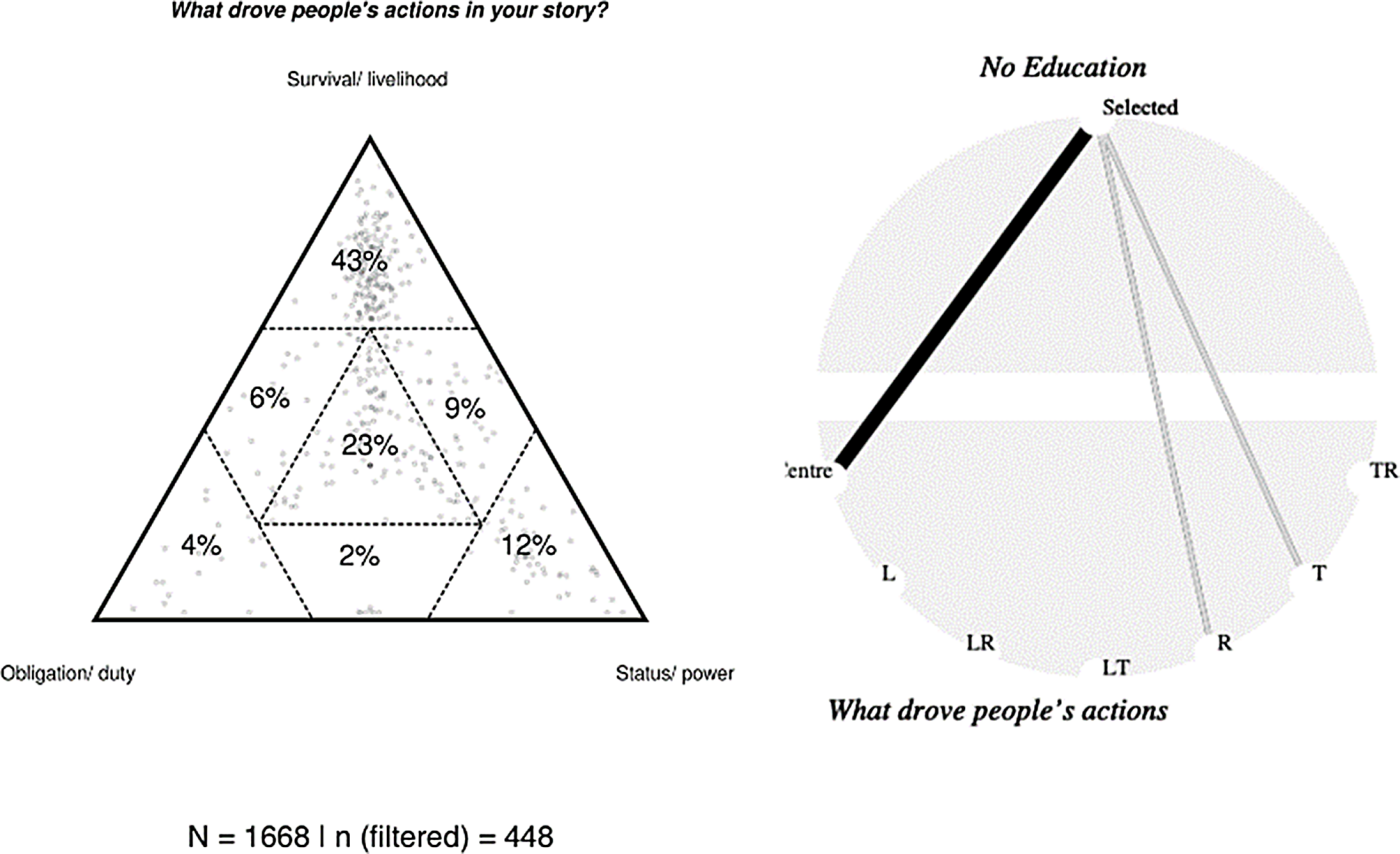
Triad 1 and cobweb diagram 1. Association between access to education and drivers of action. Triad has been filtered to display only stories where there was an inability to access education

Key patterns that were generated by SenseMaker participants’ interpretations of their own stories, were visually identified in the meta-data, and then further analyzed and contextualized firstly by the research team to identify areas for focus, and secondly in co-analysis and insight-to-action workshops with community members and then finally with institutional stakeholders. The research team compiled the data into groups of stories around particular themes based on meta-data pattern analysis - also called “storybooks” for community members to analyze and discuss. A secondary analysis was conducted focusing specifically on the theme of food insecurity which emerged as a key issue for participants. SenseMaker data were viewed especially through the lens of the multiple-choice question that gave participants the opportunity to select whether food insecurity, defined as ‘not having enough food’, was influential in their story. The patterns of responses to this question were compared across different participant demographics and other identifiers. Meta-data patterns, created by the participants’ responses to the signification framework concepts, were also compared for different experiences of deprivation. Analysis focused mainly on one concept of the signification framework which looked at drivers of action in the story (Fig. 2, Triad 1). Mentions of “lack of food”; “not enough food”; “struggle to provide food”; “hunger” or “starvation”; “not enough food to eat”, and similar references among qualitative data, were interpreted as indications of food insecurity.

Correlation analyses were carried out using Pearson’s goodness-of-fit chi-square tests [27, 28]. These show us how the frequencies we observe in the data, whether it is frequency of stories in an area of the triad-the name of a visual type of question- or the frequency of response to a question, differ from the frequencies we would have expected if our variables were independent, when we compare across categories. The results of the correlation analyses are depicted as “cobweb diagrams” (see Fig. 2, cobweb diagram 1), where each connection indicating good chances of variation from independence is depicted as a line and color-coded black for a positive correlation and grey for a negative one. The numerical value of the residuals squared for these associations has to be greater than 4, a number indicating a greater departure from independence than would be expected for 95% of the variables. The connection is depicted between the factor being compared and the relevant area of the triad, when T indicates top, R the right-hand corner, and so on. The thickness of the line indicates the strength of the correlation, which is also expressed numerically as discussed in the results, and usually accompanied by a p-value, indicating the significance level. Significance testing was also carried out to facilitate comparison between groups, comparing triad means for each group and determining whether the difference is statistically significant. For all such comparisons, p values based on non-parametric Hotelling t-tests are provided.

#### Ethics

The study protocol was reviewed and approved by the Medical Research Council of Zimbabwe and the Research Council of Zimbabwe (reference number: MRCZ/A/2594), and Allendale Investigational Review Board (reference no: IRB-WRC0001). The study procedures were also approved by the Zimbabwean Ministry of Public Service, Labour and Social Welfare (reference number: SW 8/26). The research team obtained informed written or verbal consent and assent prior to all data collection activities. For participants below age 18 who were married, living in a household without a guardian, or living in a household with a guardian but childbearing, informed consent was obtained. Participants were informed of the purpose of the activities and were assured that their decision to participate or not participate in the study would not affect their eligibility to receive services or involvement in any Plan International Zimbabwe programs. Data were anonymized and stored securely on password-protected devices only available to the research team.

## Results

### Participant characteristics

A total of 22 staff participated in 17 KIIs (12 female, and 10 male). Ten were based in Harare, of whom six staff worked for INGOs and four participants worked for UN agencies. Among the 12 participants based in Chiredzi, 9 participants worked for NGOs and CBOs, with one participant from a government ministry and one participant from a health service provider. For three interviews, more than one staff person from a given organization participated in the interview.

Across the study sites, 1,668 community members participated in SenseMaker data collection activities. The majority of participants (n = 1,098, 66% of the sample) were female compared to male (n = 570 participants, 34%). Slightly more adults (n = 954, 57%) compared to adolescents 10–19 (n = 714, 43%) participated. Only 7% (n = 48) identified as married adolescents (defined as either self-reported married or co-habiting), of whom 43 married girls and 5 married boys. Approximately 85% (n = 605) of adolescents identified as single, while 16 were divorced (all girls); one was engaged, 25 were in a relationship, and 19 preferred not to disclose their relationship status. In addition, 58 adolescent girls (14% of adolescent girls) were mothers (see Table 1).

**Table 1:** Participant demographics by research method

	Women/Girls	Men/Boys	Married^a^	Unmarried^b^	Adults (20 + years)	Adolescents (10–19 yrs)				Total
**SenseMaker**						10–12	13–14	15–16	17–19	
	1,098	570	857	789	954	56	151	344	163	1,668^c^
**KII**	12	10	N/A		22	/	/	/	/	22
	1,110	580	857	789	976	714				1,690

^a^SenseMaker married category includes 578 married, 5 engaged, 123 widowed, 140 divorced participants and 11 cohabiting

^b^SenseMaker unmarried category includes 739 single, 50 in a relationship

^c^Includes 22 participants who preferred not to disclose their marital status

### Food insecurity was a primary concern among community members

Food insecurity was described as a component of poverty and unmet basic needs, rather than as an isolated factor. When community members were asked what issues people experienced, food insecurity was the primary concern regardless of gender and age. Food insecurity is present in almost half (48%; n = 729) of stories (n = 1668) alongside other factors such as unstable income and/ or inability to access education (see Table 2).

**Table 2:** People identifying the presence of a challenging experience in their stories according to age and gender – by % of stories

Challenge in story:	10–14	15–19	20–29	30–39	40–49	50+		Male	Female
Food insecurity	31.8	30.2	33.4	32.8	28.4	29.6		30.8 (n = 241)	31.3 (n = 488)
Income insecurity	25.8	23.6	27	24.3	20.6	24.9		25.6 (n = 20)	23.9 (n = 372)
Dependents	4.2	7.1	7.1	6.2	6.5	5.4		5.6 (n = 44)	6.8 (n = 106)
Education access	22	22.7	18	19.5	21.3	24.5		21.6 (n = 169)	20.9 (n = 326)
Health access	1.7	2	3.5	2.1	5.8	1.4		2.3 (n = 18)	3 (n = 46)
Housing	2.5	5.3	6.9	6.2	9.7	2.5		4.9 (n = 38)	6.2 (n = 97)
COVID-19 mobility restrictions	6.8	6	2.5	5.3	4.5	7.6		5.4 (n = 42)	5.1 (n = 80)
COVID-19 more broadly	5.1	3.2	1.5	3.6	3.2	4		3.8 (n = 30)	2.8 (n = 44)

Statistically, correlations with the entirety of the question of deprivation are difficult to present, since participants were allowed to select as many options as desired. We will therefore focus on associations specifically with the parameter of food insecurity given the focus of this paper. Limited access to food is the most common indicator of deprivation present in the stories shared by both adolescents and adults, but it is slightly more frequent in adults’ stories (X^2^ (9, N = 1668) = 28.35, p = 0.00). Specifically, there is a negative association with food insecurity for those aged 10–12 and 15–17, and a positive one for those aged 26–29 and 30–39. Similarly, although it remains the most frequently cited experience of deprivation across locations, it appears more frequently in Chiredzi urban compared to the peri-urban study site (X^2^ (1, N = 1668) = 11.19, p = 0.00). Among those who indicated food insecurity was present in their stories, 42% (n = 241) were male and 41% (n = 448) were female, a rate similar to that of the overall sample size, indicating no gendered difference in that aspect.

### Food insecurity elevates girls’ risk of child marriage

**Fig. 3:**
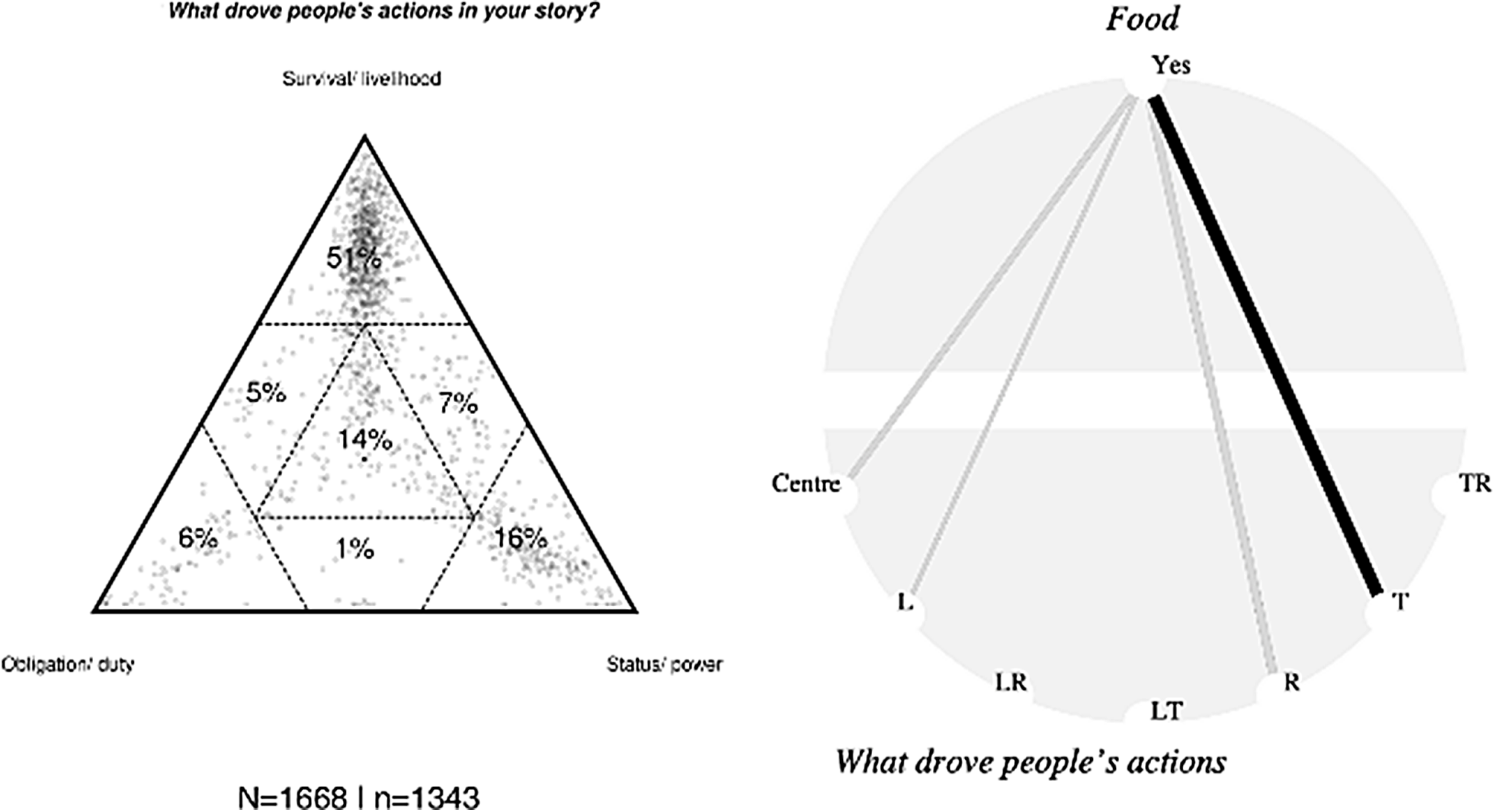
Triad 2 (left) and cobweb diagram 2 (right). Association between food insecurity and drivers of action

Correlational analysis (Fig. 3, X^2^ (6, N = 1343) = 64.86, p = 0.00) of the SenseMaker data shows that where participants indicated that food insecurity was present in their stories, survival/livelihood (T) was more likely to be a driver in peoples’ stories about marriage (triad 1, pictured in Fig. 3 for all respondents). The comparison of participants’ responses for stories where food insecurity had been present with stories where it had not (see Fig. 3) indicates that the influence is highly statistically significant t(6 = 40.91, p = 0.00). The high concentration of stories aligned with food insecurity in the top portion of the triangle indicates the importance of acting out of necessity as underpinning agency and action in relation to marriage.

Earlier, we identified inability to access education as a factor that participants partially see as different from food insecurity. This is corroborated by Fig. 2, which shows patterns of stories associated with inability to access education.

As the cobweb diagram 2 indicates (X^2^ (6, N = 1343) = 76.30, p = 0), in stories where there was particular emphasis on inability to access education, there were more stories placed at the center of the triad, while there were fewer stories placed at the top of the triad (T). Although survival is still considered to be the strongest driver in stories, there is much greater awareness of the combined effect of multiple drivers, giving us a hint of how overwhelming and single-focus the literal survival pressure of food insecurity can be.

### The influence of food insecurity on decision-making practices in the context of the social norms framework

Stories from adolescent girls and adult community members indicate that decision-making pathways between food insecurity and child marriage are underpinned by gender inequality and socio-economic inequality and poverty. Within these social and cultural affordances, perceptions of decision-making and agency differ between adolescent girls and caregivers. Parents might perceive or be forced to turn towards child marriage as a means to ameliorate the economic burden of caring for their daughters and their unmet needs, such as limited or lack of food in the household. The perceived economic burden of daughters and unmet needs within a context of abject poverty and gender discrimination, elevate girls’ risk of child marriage by rationalizing caregivers’ decision to exert their power to force their young daughters into marriage; encourage their daughters to marry; or passively accept their daughter’s decision to initiate her own marriage. Food insecurity elevates girls’ risk of initiating her own child marriage because she might perceive marriage as a means to escape abuse and unmet needs, including limited or lack of food, in her home.

Stories about child marriage told by adolescent girls, largely demonstrated that adolescent girls’ perceptions about marriage outcomes influence their decision-making. In the following story, an adolescent girl shares that her peers perceive marriage as a means to escape food insecurity in their homes.

Sometimes the family will be facing food shortages that even the father sleeps on an empty stomach, in fact the whole family will be hungry because the family will be poverty-stricken. It’s not that the family will be withholding food from their child deliberately; on the other hand, the child may perceive it the other way and opt to get married as a solution out of poverty and abuse. Adolescent girl, 18–19 years, single, with children, Chiredzi Urban.

#### Adolescent girl and boy decision making about child marriage are impacted differently by food insecurity

**Fig. 4:**
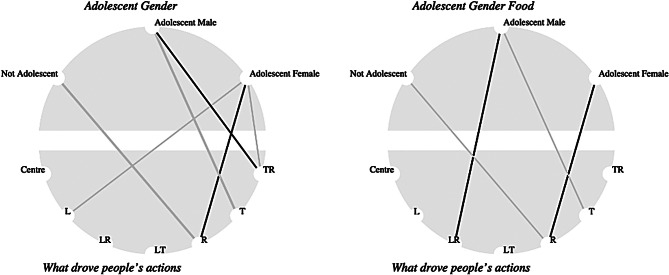
Association between drivers of action and gender

Stories shared by adolescent girls as well as adult community members, indicate perceptions that adolescent girls living in food insecure households are at an elevated risk for child marriage. Correlational analysis on the triad around drivers of action indicates differences between adolescent girls and boys on this topic. Regardless of whether food insecurity was said to be present, adolescent girls associated actions being driven by status/power more than other groups (R, see Fig. 4 X^2^ (12, N = 713) = 24.02, p = 0.02). Whereas for adolescent boys there is a stronger association with obligation/duty as a driver for action in combination with status/power (LR) when food insecurity is present (X^2^ (12, N = 713) = 4.78, p = 0.020188). Where food insecurity was indicated by participants as not being explicitly present, adolescent girls were less likely to identify obligation/duty (L) as a driver in the stories they shared. Adolescent boys in the overall dataset more strongly associated drivers with a combination of survival/livelihood and status/power (TR, see Fig. 4, X^2^ (12, N = 713) = 33.781, p = 0.00). Taken together, these findings quantify some of the notions around gender roles and perceptions of marriage.

It is important to reiterate that the primary driver of actions for all groups is survival, and we are exploring the fluctuations of other drivers and their combinations, with the understanding that survival remains primary. According to community gender norms seen in the stories, community co-analysis sessions^1^, getting married and being “provided for” by a husband and maintaining the home are essential parts of a woman’s role. According to a participant in the community co-analysis sessions:

Girls in ward 4 [Chiredzi urban] do not even think about getting a job. The norm is that men should work and provide for their families. It does affect what they do later in life, because for them sometimes they just think that ‘I am a girl and at the end of the day I will get married.’ They may then not concentrate much on their education because the end-goal is marriage. They tell themselves that ‘At the end of the day, I’ll still get married, I will become a mother, I will stay with my husband who should then take care of me.’ Hence the high number of men who migrate to neighboring South Africa in search of jobs. If a man is not employed and spends most of his time at home, the community will laugh at them saying that he was henpecked [given a concoction of ingredients to be made docile]. Married man, Chiredzi Peri-Urban adult co-analysis participatory discussion.

Given these gendered expectations, as well as the shortage of life alternatives to marriage (see later section on sexual exploitation), it is unsurprising that survival is associated with a drive for status or power by adolescent girls. Although the SenseMaker numbers suggest that women are participating as much as or more than men in income-generating activities (34% of women vs. 30% of men in the sample regularly working), a lot of this work can be experienced as precarious, and less desirable than what marriage provides, within existing gender norms. The perspective quoted above also illustrates the community pressure faced by young men and boys to be able to support a family (“take care of me”, above), a pressure that manifests in their greater emphasis on obligation, a theme repeated in stories.

#### COVID-19 and climate hazards exacerbated food insecurity and child marriage

Stories indicate that food insecurity was among the factors of deprivation that pushed parents to marry their daughters to relieve resource constraints in the home due to economic instability and poverty engendered by COVID-19 and climate hazards.

When KIs discussed the consequences of COVID-19 on child marriage, food insecurity emerged more strongly as a factor that pushed parents to marry their daughters. Due to economic hardship engendered by COVID-19 containment measures, KIs and a few SenseMaker stories suggest that both parents and adolescent girls may have perceived child marriage as a means to ameliorate food insecurity for the first time during COVID-19. A KI explains:

People’s livelihoods were disturbed a lot. So, some of the families encouraged their girl children [to marry] and the children themselves have seen that there is a lot of hunger at home and [they think] ‘it is better [that] I accept this man’s proposal and be married so that I can be helped as he looks like he has money’. KI, government official.

To a lesser extent, food insecurity as a consequence of climate hazards (e.g., flood, droughts, extreme heat) was discussed as a key concern facing families and a reason why parents force their daughters into child marriages. The following NGO staff person explains:

…The drought also impacted child protection, especially child marriage, where families in a situation of food insecurity… start pushing out girls to get married as a means of trying to ease the economic burden by lessening the number of people to feed in the household. Marrying off girls is an easy option and the family in turn gets lobola [bride price], which makes child marriage an opportunity for them to get a bit richer while also lessening the number of people to feed in the household. KI, NGO staff person.

#### The role of migration to South Africa on child marriage

Child marriage also emerged in qualitative data and participatory analysis discussions as an unintended consequence of parents’ migration to South Africa in search of work due to factors including food insecurity and lack of employment opportunities, leaving adolescents with reduced support, guidance, and resources. Although stories emphasized lack of parental guidance and control as the dominant factor resulting in child marriages (and adolescent pregnancies) among child-headed households, deprivation of unmet needs, such as references to “starvation”, also emerged. In the following story where food scarcity was present, a woman in Chiredzi peri-urban explains how parental migration to South Africa resulting in child-headed households may lead to child marriage:

Where I live there are girls who get married below the age of 17 years. The reason behind we don’t know. Maybe it’s the loss of parents, or starvation but we honestly don’t know what pushes them to do those things. Sometimes we think they are possessed. Their parents do not even care. They [parents] go to South Africa leaving their children without no one to look after them. These young girls take over the house into their own hands and brings her boyfriends into her mother’s house. Woman, 20–22, married, Chiredzi Peri-Urban.

Being orphaned (by parents’ death rather than migration) also seemed to spur child marriage. Orphans’ lived experiences were portrayed as needing material (e.g., food, clothing, school fees), social, and spiritual resources, occasionally resulting in marriage as a recourse:

Absence of caring relatives when children are orphaned is a contributing factor in most cases where 10-year-old children marry each other. Relatives or well-wishers who can give out food parcels. People just talk and do not help in most cases even with a small cup of mealie meal. Man, 40–49, divorced, Chiredzi Peri-Urban.

Migration of men and older adolescent boys to South Africa emerged as a subtheme related to girls’ decision-making pathway toward child marriage. Similar to parents, qualitative findings indicate that men and adolescent boys migrate to South Africa in search of work due to factors including food insecurity and income generating opportunities. Due to socio-economic inequality, lack of alternative opportunities to marriage, and pressure to conform to gender roles, many adolescent girls aspire to marry *Joni Joni’s*^2^ (Zimbabwean boys and men migrating to work in South Africa), because they are perceived as more financially stable than males living in Chiredzi and a pathway to establish a better life in South Africa.

Upon their return to Chiredzi, *Joni Joni’s* may participate in the normative practice of child marriage in a position of power and privilege in the community. A KI explains:

They [communities in Chiredzi] view marriage as an asset, so it’s an aspiration for girls to get married and also for boys it’s also an aspiration to get married…. even for the adult men. It’s something which they value quite well and it’s also an aspiration to get with someone who has crossed the boarder… and he comes after a year at Christmas, and he brings stuff for the home or for them when they get back. That’s how I see it here. KI, government official.

#### Education may be a modifier in the pathway to child marriage

While food security among other factors of deprivation, such as unstable/lack of income, drive child marriage, education may act as a modifier that reduces girls’ risk of marriage.

Associations among food insecurity and other factors of deprivation are complex and varied. Participants who stated that there was a lack of sufficient food in the story shared were much less likely to select that there was also an inability to access education than those who did not indicate lack of sufficient food. Conversely, there was a positive association between food insecurity and unstable or no income (the residual squared = 83).^3^ Without denying the intersections of indicators of deprivation, we should recognize that to participants’ experiences and perceptions, these categories are not monolithic.

While lack of school fees is often, but not always, mentioned in isolation from other factors of deprivation in the lack of education storybook, food insecurity is always mentioned in conjunction with at least one other factor of deprivation, such as lack of safety in the home and abuse:

There is a girl who got married. She was being abused by her grandmother, they would argue. She started bringing boyfriends home and when her grandmother reprimanded her, she resolved that it was better to get married, at that time she was 14 years old. -Chiredzi Peri-Urban, Female, 10–12, Single.

Indeed, there were many stories that mentioned lack of school fees and school dropout as an impetus for child marriage in the food insecurity storybook, whereas references to food insecurity were far less common in the lack of education storybook. This observation supports the finding above education may be a modifier of child marriage; while food insecurity is one of several intersecting factors that compound child marriage.

## Discussion

This study documented the complex linkages between food insecurity and child marriage among food insecure communities in Zimbabwe. We found that food insecurity was among the contextual factors of deprivation that influenced parents’ and adolescent girls’ decision making around child marriage. Findings align with grey literature that suggest that caregivers marry off their daughters as a coping strategy to lower resource burdens or to have one less family member to feed [29–32]. This study demonstrates that although food insecurity was the primary concern among all community members regardless of sex or age, it is not an independent driver of child marriage. Instead, food insecurity intersects with other factors across levels of the Social Norms Framework for Child Marriage in Crisis (institutional, individual, social, crisis) that influence child marriage decision making. Above all, decision making (power) is grounded by social and gender norms entrenched within communities that systematically undervalue girls. This, coupled with conditions of food scarcity further limits both decision making power, but also the potential to open new possibilities and make change for adolescent girls and young women.

As illustrated by the Social Norms Framework for Child Marriage in Crisis, the study also reveals varying motivations that lead to child marriage, which suggest deeper social and gender norms are at play and exacerbated in conditions of poverty and resource scarcity, including food insecurity. Stories present multiple perspectives that illustrate these interrelated perceptions including the hope many adolescent girls attach to men and marriage, namely that it will liberate them from conditions of poverty and/or abuse. As such, girls themselves are initiating their own marriages; however, these choices are made in contexts of limited alternative survival opportunities and within the restraints imposed by community norms around acceptable gender roles, food insecurity, poverty, abuse, and parental migration. Findings contribute to global literature that some adolescents are exercising their (arguably limited) agency and choosing to marry against a background of extremely limited options [20, 32, 33]. Child marriage decision making is underpinned by unequal power dynamics that discriminate against adolescent girls’ well-being [31]. Although community members largely relay stories about girls entering into relationships, including marriage, in a tone of disapproval, adolescent girls perceive marriage as the only or best alternative to the hardships they face at home and limited employment opportunities in Chiredzi. In this way, adolescent girls are taking action to survive, and many aspire to change their life trajectory for the better.

Although the positive impact on engaging adolescents boys (and men) in health promotion and violence prevention for women and girls is established in the evidence-based, [34] adolescent boys’ perspectives on child marriage decision making is less clear. This study presents differences in how food insecurity in particular influences adolescent girl and boy decision making pathways to child marriage, which align with gender norms in the community. When food security was present in adolescent boys’ stories, there was a stronger association with obligation/duty as a driver for action in combination with status/power, which indicates the heightened burden that adolescent boys possess to fulfill the gendered role of being the sole provider of the family. However, more research is needed to understand adolescent boys’ perspectives of child marriage to effectively engage them in prevention approaches.

Due to the dominant theme of GBV against girls perpetuated by parents and others in the home, all humanitarian actors must work together with national counterparts, namely feminist and women-led organizations, to implement gender transformative programming that challenges communities to critically reflect on social and gender norms and amend harmful practices that reduce the role of women and girls in society. Social protection services delivered by national or international actors should also ensure that existing community child protection mechanisms and case management systems are functional and responsive to the needs of adolescent girls.

Consistent with evidence in other humanitarian and development contexts, education emerged as a promotive factor in the prevention of child marriage [35–37]. In contrast, lack of access to education, including barriers to education such as lack of school fees or school materials, was associated with child marriage. Therefore, multi-sectoral prevention programs may consider including approaches to keep girls in school by mitigating access barriers, such as household cash transfer initiatives conditional on daughters attending school. Such programs have the potential to mitigate the social and economic drivers of child marriage; [38] however, they should be sensitive and responsive to local child marriage practices and drivers. School feeding programs are the primary source of nutrition and sustenance for most children and adolescents in Chiredzi. Given study findings indicate an increase in food insecurity leading to child marriage during COVID-19 when schools were closed, schools should extend their feeding program for students outside of operating hours-including during school holidays-and extend the program to out of school adolescents to mitigate food insecurity in the home and prevent child marriage.

Overall, findings suggest that gender transformative and community-led child marriage programming is needed to mitigate the drivers and consequences of child marriage before, during, and after humanitarian crises occur. It is therefore essential that during a crisis response humanitarian actors ensure that pre-existing programming and services to tackle child marriage are maintained; and that mitigations are put in place across the delivery of aid, especially food aid, and that intentional actions are taken to mitigate the exacerbated drivers and risks of child marriage girls and their families face as a result of the crisis [32]. Community-led humanitarian programming provides (a) an opportunity for adolescents to participate in decision-making, and (b) ensures interventions are premised on a deep contextual understanding of marriage practices informed by the perceptions of multiple stakeholders. Better involvement of the community, including adolescents, in the development and implementation of programming results in more tailored services and helps to preempt unintended consequences.

Limitations to study findings exist. First is the issue of temporality. Participants’ shared stories about child marriage in their community which may be reflective of a near recent experience, or of the past. Therefore, we cannot make strong causal links between food insecurity and the qualitative data presented in this paper. Second, findings are subject to social desirability bias. Community member participants may have underreported their marital age out of fear of repercussions given that child marriage is illegal in Zimbabwe. This may be one reason why our sample size for married adolescents was small. Third, given our small sample size for married adolescent boys, these findings largely focus on the child marriage experiences of adolescent girls. Additional research is needed to understand the risks and drivers of child marriage among adolescent boys in food insecure settings. Finally, the participatory design and analysis of SenseMaker data among community members, research teams, and Plan International Zimbabwe program staff required more staff time than anticipated. More time should have been planned and budgeted for comprehensive training on the methodology and for analysis of the data.

Despite these limitations, to our knowledge, this is the only community-based and girl-centered study aimed at understanding linkages between child marriage in food insecure settings, and findings have critical implications for humanitarian programming, particularly as food insecurity is expected to increase as a result of climate change [7]. By inviting participants to analyze their own story, SenseMaker prioritized participants’ views within the analysis. This approach both (a) lessened interpretive and cultural bias; and (b) ensured that respondents’ perspectives were central to the research process. By asking participants to share stories widely about what it is like for young people to grow up here, the qualitive data illuminated the more tacit or taken-for-granted elements of gender norms that can often not be stated explicitly, via the multi-narratives from community members of different ages, genders and other diverse characteristics such as marital status. Findings also have implications for development actors who should consider strengthening girls’ access to education and nutrition programming in preparedness and disaster risk reduction frameworks.

## Conclusion

Study findings illuminate that food insecurity is among other factors of deprivation that increase girls’ risk of child marriage. Food insecurity factors strongly in parent’s decision-making to force or encourage their daughter to marry, and when adolescent girls initiate their own marriage to meet their needs. In contrast, the study found that education may mediate girls’ risk of child marriage. This research suggests that child marriage programming should be community-led and gender transformative to address gender inequality that underpins child marriage (see Fig. 1). Further, child marriage programming must be multi-sectoral to address the intersecting levels of deprivation and elevate the capacities of adolescent girls, their families, and communities to prevent child marriage.

### Electronic supplementary material

Below is the link to the electronic supplementary material.

**Supplementary Material 1**: SenseMaker tool

**Supplementary Material 2**: Key informant interview guides

## Data Availability

The data were collected in food insecure communities and included sampling married adolescents and discussing child marriage. Given these sensitivities, de-identification and posting to a repository while maintaining the content is challenging. Therefore, data may be made available upon request to the corresponding author.

## List of abbreviations

Chiredzi: Chiredzi District
CBO: Community-based organization
CP: Child Protection
CSO: Civil society organization
GBV: Gender-based violence
IPV: Intimate Partner Violence
IPC: Integrated Food Security Phased Classification
INGO: International non-governmental organization
KII: Key Informant Interview
NGO: Non-governmental organization
Plan: Plan International
UN: United Nations

## Footnotes

1 In October 2021, the research team facilitated participatory community analysis discussions with adults and adolescent girls in Chiredzi urban and Chiredzi peri-urban. The purpose of the discussions was to provide an opportunity for adolescent girls and adult community members to analyze the data through participatory group activities and identify girl-led and community-grounded solutions to child marriage. A final participatory data analysis discussion was held with institutional stakeholders.

2 A nickname for adolescent boys and young men derived from Johannesburg, the city in South Africa where they are generally assumed to be based.

3 The value indicates the squared residual of the Pearson’s Chi-Squared test for this association, with a count above 4 indicating a value significantly different from random chance.
