# Supplementary material for: Associations between child marriage and food insecurity in Zimbabwe: a participatory mixed methods study
Source: BMC Public Health. 2024 Jan 2;24:13. doi: 10.1186/s12889-023-17408-7 (PMC10759370; doi:10.1186/s12889-023-17408-7)
Supplement: Supplementary file 1 — Supplementary Material 1: SenseMaker tool [file 12889_2023_17408_MOESM1_ESM.docx]

**Annex 1. SenseMaker Tool (English)**

**1.1** **Think about young people getting married in your area.** ​ **Can you share a story**​ **about what it’s like for a young person in your community to be married? When we say young person, we mean a person who is 10-19 years old.** The story can either be​ from your own experience or one that you know about.

**1.2 If you were to describe this situation in a few key words or give it a title, what would they be?**

|  |
| --- |

Next are some statements to help reflect on your story. Please move the ball in the triangle to the position that best describes the story you have just shared. You can place the ball anywhere in the triangle. The closer you are to each corner, the stronger this statement is for you. If all three apply equally, you can place it in the center. If a statement is not relevant or applicable for your or experience, you can click the N/A box.

**Below is an example to help illustrate how this type of question works. Move the ball around to indicate how you like your plate of sadza in the evening . If you don't like sadza at all, you can click N/A.**

| **2.1 How do you like your plate of sadza?** | |
| --- | --- |
| Sadza | |
| 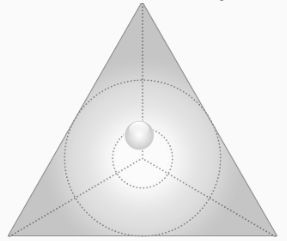 | |
| Sauce/soup | Meat |

The following questions ask about the story you just told.

| **2.2 What drove peoples’ actions in your story?** | |
| --- | --- |
| Survival/ livelihood | |
| 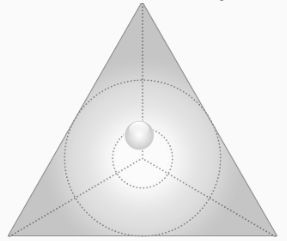 | |
| Obligation/ duty | Status/ power |

| **2.3 Who or what is responsible for what took place in your story?** | |
| --- | --- |
| The individual / me | |
| 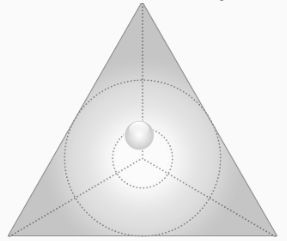 | |
| Culture/tradition | People with power and authority |

| **2.4 What happened in my story was influenced by…** | |
| --- | --- |
| Young people, 10-19 years of age | |
| 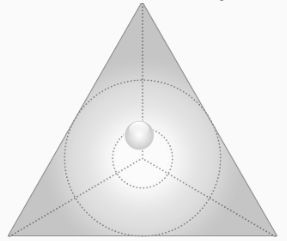 | |
| Family/ immediate community | The outside world |

| **2.5 In my story people….** | |
| --- | --- |
| Had the motivation to do it | |
| 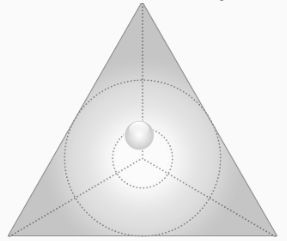 | |
| Knew what to do | Felt supported to make it happen |

| **2.6 Things could have been better with….** | |
| --- | --- |
| Knowledge and understanding | |
| 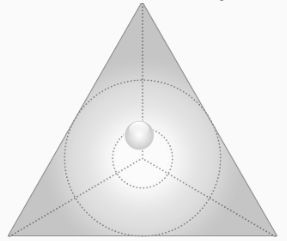 | |
| Services (health, education, livelihoods) | Community support |

Please now move the marker on the sliding scales below to a position that best reflects the story you told at the beginning. Think about how your story sits between these two points, and the closer the marker is to an extreme corner, the stronger the pull of this is for you. If in doubt, go with your gut feel placement. Remember to answer based on the story you just told.

**3.1 In my story, peoples’ attitudes were about…**

| Keeping  tradition | 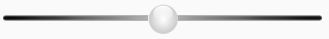 | Changing life as we know it |
| --- | --- | --- |
| **3.2 Any inform** | **ation in my story was…** |  |
| Too much, overwhelming | 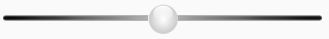 | Not enough, or not helpful/ appropriate |

**3.3 In my story, people were…**

| Totally alone | 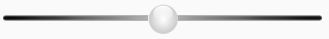 | Aware that others have been or are in a similar situation to me/them |
| --- | --- | --- |

**3.4 People in the story were...**

| Completely stressed or overwhelmed | 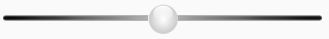 | Totally carefree |
| --- | --- | --- |

**Below are a few more questions about the story you shared earlier. Unless instructed otherwise, please choose one response option only.**

**4.1 The experience you described is…**​ (Select only one response)

◻A one-time occurrence

◻Rare

◻Somewhat common

◻Very common

◻Not sure

**4.2 How do you feel about your story?**​

◻Very good

◻Good

◻Neither good nor bad

◻Bad

◻Very bad

**4.3 How quickly did things happen in your story?**

◻So quickly there was no time to think

◻Quickly

◻Average / neither quick nor slow

◻Slow and controlled

◻Much too slowly

**4.4 Who should hear about your story? (Select all that apply).**

◻Only me

◻My mother

◻My father

◻My grandparent(s)

◻My aunt/uncle

◻Other relatives

◻Community leader ◻Traditional leader

◻My friends

◻My teacher

◻My religious leader

◻My community leader

◻Health/social worker

◻Government official

◻International agencies

◻Other______ please specify

**4.5 Who had the most influence over any decisions made in your story?**

◻Only me

◻My mother

◻My father

◻My grandparent(s)

◻My aunt/uncle

◻Other relatives

◻Community leader

◻My friends

◻My teacher

◻My religious leader

◻My community leader

◻Traditional leader

◻Health/social worker

◻Government officials

◻International agencies

**4.6 Trusted knowledge in your story comes from… (choose up to 3)**

◻Mother

◻Father

◻Family members, e.g. aunts/ uncles

◻Friends

◻Religious or civic leaders in the community

◻Official guidelines of education

◻Social media (e.g. Facebook)

◻Personal research ◻Other:

◻_________

◻N/A

**4.7 What was the living situation in your story? (Select all that apply)**

◻Children or young people living with both parents

◻Children or young people living with one parent

◻Children or young people living with no parent

◻Child or young person living with their partner

◻People were chased out of their home

◻People immigrated to/from Chiredzi to earn money and have a better life

◻People were displaced

◻Other situation

**4.8 At the time of the story, did the person in the story experience any of the following? (select all that apply)**

◻Not having enough food

◻Very unstable or no income/financial support

◻Needing to take care of people depending on you

◻Inability to access education for any reason

◻Inability to access health services

◻Bad quality or no housing

◻Restricted mobility as a result of COVID-19

◻Other COVID-19 restrictions

◻ COVID-19 related sickness in the family

◻ None of these

**4.9 Do you think COVID-19 influenced your story in any way?**

◻COVID has nothing to do with my story

◻The story happened during the COVID-19 pandemic, but it had no impact on how things happened in my story

◻COVID played a big part in how things happened in my story

◻No response

**4.10 When thinking about the next generation, I would like to see…**

◻More stories like the one I’ve shared

◻Less stories like the one I’ve shared

◻Not sure

**Below are a few questions about YOU. Please choose one response option only.**

**5.1 [Enumerator: select gender of participant]**

◻Male

◻Female

**5.2 How old are you? [one response only]**

◻10-12

◻13-14

◻15-17 ◻18-19

◻20-22 ◻23-25

◻26-29

◻30-39

◻40-49

◻50+

**5.2 What is your religion?**

◻ Christianity

◻ African Traditional Religion (indigenous)

◻ Islam

◻ None

◻ Prefer not to say

- 1. **What ethnic group do you belong to?**

◻​Shangaan​i

◻​Shona​

◻​Venda​

◻​Kalanga​

◻​Ndebele​

◻ None of these

- 1. ​**Are you currently in school?**_​_

◻Yes

◻No

- 1. **What is the highest level of education you have completed?​ Choose the best response.**

◻ Some primary school

◻Completed primary school

◻Secondary school

◻Ordinary level

◻Advanced level

◻Other

◻No school

**5.6**​ **Are you currently working to generate income?**_​_

◻Yes

◻Sometimes/seasonally

◻No

**5.7** ​**If yes, what is your primary source of livelihood?**_​_

◻Seasonal farm labor

◻Subsistence Farming

◻Artisanal mining

◻Domestic work

◻Vending

◻Professional (e.g., teachers, nurses, bankers)

◻Self-employed (e.g., dressmaking, carpentry)

◻Other

◻Not employed/ N/A

**5.8 What is your marital status?**

◻Single

◻In a relationship

◻Cohabiting

◻Engaged

◻Married

◻Divorced

◻Widowed

◻Prefer not to answer

**5.9 [If ever married], at what age were you first married?**

◻Under 10

◻10-11

◻12-14 ◻15-17 ◻18-19

◻20-24

◻25 or above

◻N/A [never married]

**5.10 [For girls and women only] Have you ever given birth?**

◻No

◻Yes

◻Prefer not to respond

◻N/A (For boys and men)

**5.11 How old were you when your first child was born?**

◻------ OPEN

◻N/A

**5.12 How many children do you have?**

◻------ OPEN

◻N/A

**Thank you. The next questions ask about difficulties you may experience because of a health problem.**

**5.13 Do you have difficulty seeing, even if wearing glasses?**

1. No-no difficulty
2. Yes-some difficulty
3. Yes-a lot of difficulty
4. Cannot do it at all

**5.14 Do you have difficulty hearing, even if using a hearing aid?**

1. No-no difficulty
2. Yes-some difficulty
3. Yes-a lot of difficulty
4. Cannot do it at all

**5.15 Do you have difficulty walking or climbing steps?**

1. No-no difficulty
2. Yes-some difficulty
3. Yes-a lot of difficulty
4. Cannot do it at all

**5.16 Do you have difficulty (with self-care such as) washing all over or dressing?**

a. No-no difficulty

1. Yes-some difficulty
2. Yes-a lot of difficulty
3. Cannot do it at all

**5.17 Do you have difficulty remembering or concentrating?**

1. No-no difficulty
2. Yes-some difficulty
3. Yes-a lot of difficulty
4. Cannot do it at all

**5.18 Using your everyday language, do you have difficulty communicating, for example understanding or being understood?**

1. No-no difficulty
2. Yes-some difficulty
3. Yes-a lot of difficulty
4. Cannot do it at all

Here is one final question, which you do not have to answer if you don’t want to.

**6.1 Do you remember a moment where you stopped being a child, and became an adult? Tell us about this. (optional)**

**[Take detailed notes as participant shares his/her experience with you.]**


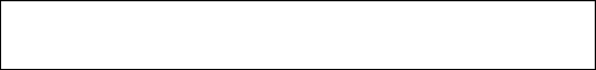


**Thank you for taking the time to share your experiences and perspectives!**

**7.0 For enumerator, share any reflections from the data collection experience (e.g., environment where the story collection took place, body language of participant, distractions, other observations) and a few bullet points about what happened in the story.**


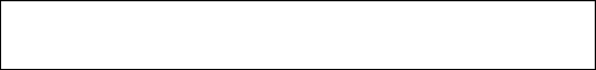


**Please hit the “Save Story” button below.**
