# Supplementary material for: Associations between child marriage and food insecurity in Zimbabwe: a participatory mixed methods study
Source: BMC Public Health. 2024 Jan 2;24:13. doi: 10.1186/s12889-023-17408-7 (PMC10759370; doi:10.1186/s12889-023-17408-7)
Supplement: Supplementary file 2 — Supplementary Material 2: Key informant interview guides [file 12889_2023_17408_MOESM2_ESM.docx]

**Annex 2. Key Informant Interview Guides**

**Key Informant Interview Guide: Local NGOs and INGOs**

*Background and Context*

First, I’d like to ask you some general questions about your experience working in this setting.

1. What is your current role and length of time in this position?
2. Do you have experience working with adolescents and their families in this role or in previous roles?
   1. **If yes**: can you tell me more about your experience?
3. How do you define adolescence? What marks the boundaries of adolescence? In other words, when do girls become women?
4. In your view, what are the most pressing issues facing adolescent girls in this setting [*define setting/location referenced, which will also frame the remaining questions*]?
   1. Are there any differences in issues faced by younger adolescents (10-14 years of age) and older adolescents (those 15-19 years of age)?
      1. **If yes**, can you tell me more about the differences?
   2. What about adolescent girls living with a disability?
5. What kinds of services or support are available for adolescent girls in this setting?
6. Which actors (e.g., CSO, NGOs, INGOs, humanitarian, government) provides these services?
7. What are the barriers faced by girls in accessing these services/support?
8. What service/support gaps exist in this community?
9. Who makes the decision for girls to participate in these services?
10. Does your [CSO, NGO] work directly with these influencers?
11. When implementing adolescent programming, what are the key challenges that you experienced in trying to reach adolescent girls?

*Marriage Practices & Programming*

1. Can you tell us about your views and insights on marriage? At what ages is marriage acceptable? Why do you feel this is an acceptable age for marriage?
2. How do you work with girls and their families to navigate marriage practices? Is this through programs (holistic or focused)? Is it through informal support?
   1. Can you describe a time when you worked with a girl and her family on the issue of marriage?
3. Now, I’d like to ask you about this community’s views on marriage. Can you tell us about how this community views marriage? At what age is marriage considered acceptable in this community? Why does the community feel this is an acceptable age for marriage?
4. Do you know of any specific programs here that address child, early, and forced marriage (CEFM)?
   1. **If yes**:
      1. Are you directly involved in such projects?
      2. Have you partnered with other agencies or organizations on CEFM programming?
      3. What are the key challenges experienced by CEFM programs in trying to reach adolescent girls?
   2. **If no**:
      1. Do you think there is an urgent need for CEFM programming here in your community? Why or Why not?
5. What challenges do you think adolescent girls face in terms of receiving information and services, such as SRH, protection, education, etc.?
   1. Are there any challenges regarding attitudes of service providers? Or program staff?
   2. How have these challenges been addressed?
6. What barriers (at the policy, community, facility, or family level) might be experienced in providing [CEFM; or sexual and reproductive health; GBV; child protection] services to adolescents in this community?
7. To what extent do CSOs/NGOs engage and work with girls, boys, families, and communities on the topic of CEFM?
8. Are there CEFM, or adolescent [sexual and reproductive health; GBV; child protection] initiatives underway, in neighboring areas that have a strong model for service delivery which could be learned from? Can you tell me what made those initiatives successful?

*Impact of COVID-19 on Marriage Practices*

1. What has been the overall impact of COVID-19 on CEFM practices in the communities where you work?
   1. Have you recognized a change in the incidence of CEFM cases since COVID-19? Increase or decrease?
      1. If yes, *how* has COVID-19 driven this change (e.g., restricted access to services/education; isolation)?
2. Have the drivers of CEFM changed? If so, how have they changed (type, magnitude, key actors etc.)
   - 1. What are the impacts of these changes in the present and what are the anticipated impacts?
3. How has COVID-19 altered decision-making pathways within households around availability and access to resources defined as human, social, and cultural capital required for healthy child development and wellbeing?

*Coordination*

1. What is your institution’s role and how does it relate to the issue of CEFM?
2. Is there a national strategy or legal framework to address the needs of adolescent girls? What are some of the key national/regional policies/laws that are relevant to the issue of CEFM for the persons affected by food insecurity in this setting?
   1. Who is reasonable for monitoring accountability on the implementation of these national/regional legal frameworks on CEFM?
   2. What about policies/laws relating to:
      1. Protection/GBV
      2. Other harmful traditional practices: FGC, and others
      3. Livelihoods/earnings
      4. Education-access of displaced to primary and secondary school
      5. Health/Education-SRH within health education curriculum
      6. Age of consent for medical services
      7. Other
3. How have different sectors (education-formal and non-formal, social service, protection, recreation and health) engaged in providing services to adolescents in this setting?
   1. Can you share specific interventions or programs underway?
   2. What challenges have been faced by such programs?
   3. Can you tell me what categories of girls (e.g., in/out of school, married/unmarried, disability) might be excluded from programs and services?
   4. Do you feel any of these programs have improved positive outcomes for adolescents as a result?
4. Who provides SRH education within this community? How is information shared with adolescent girls about the following topics:
   1. Puberty
   2. Menstruation and menstrual hygiene
   3. Fertility
   4. Communication between men and women
   5. STIs and HIV
   6. Contraception and family planning
   7. Birth spacing
   8. Pregnancy complications and warning signs
   9. Marriage
5. What child protection services are available for adolescent girls in this community?
6. To what extent do these programs address power dynamics and gender norms? Traditional practices that may be harmful or disadvantageous for young girls?
7. How are programs different by sub-populations? For example, by age group (15-19 years and 14 years and younger), marital status, gender, or disability?
8. To what extent do existing programs and services for adults and/or children extend their services to adolescent girls, and in your opinion has this approach been effective?
9. What are the differences in roles and responsibilities of women and men and boys and girls that can exacerbate risk and vulnerability?
10. What are the perceptions of women/girls and men/boys regarding their involvement in decision-making?
11. Are there differences in terms of participation and access to resources between men and women, boys and girls? What about other excluded groups such as Persons With Disabilities (PWDs) and other minority populations?

Recommendations for Adolescent Programming

1. What information would be helpful to know about the adolescent girls, in order to provide services which meet their needs?
2. How do you think evidence about the drivers of CEFM and protective assets/capacities of adolescent girls can be used to inform services or programs?
   1. Are there other organizations (e.g., advocacy, programming, or research), who might be particularly interested in this research?
   2. What other data would be helpful to gather in order to develop a program to prevent and respond to CEFM in this setting?
3. What recommendations do you have to strengthen/promote actions to prevent or stop CEFM?
4. What are the key areas that you think need to be addressed and how?
5. What would next steps be to accomplish this? What committees/government/community actors would need to be involved and in what ways?

*Closing*

Thank you so much for taking the time to answer these questions. We very much appreciate your time, as your input will help to shape this research effort going forward. Is there anything else you’d like to add about the topics we discussed?

**Key Informant Interview Guide: Faith Leaders**

*Background and Context*

First, I’d like to ask you some general questions about your role in this setting.

1. Can I start by asking a little about yourself? What is your designation/position/role in the community? What is your current role and length of time in this position?
2. How do you define adolescence? What marks the boundaries of adolescence? In other words, when do girls become women?
3. In your view, what are the most pressing issues facing communities in this setting?
   1. What about for adolescent boys? Adolescent girls?
   2. What about adolescent girls living with a disability?
4. How does your role as a religious leader relate to addressing some of the issues affecting adolescents?
5. Do you think there is an urgent need for adolescent girl programming here in your community? Why or why not?
6. What kinds of services or support are available for adolescent girls in this setting?
   1. Are you directly involved in such projects?
   2. Have you partnered with other agencies or organizations on adolescent girl programming?
7. What are the barriers faced by girls in accessing these services/support?
8. Which actors (e.g., CSO, NGOs, INGOs, humanitarian, government) provides these services?
9. What service/support gaps exist in this community?
10. Who makes the decision for girls to participate in these services?
11. As a religious leader, can you tell us about your views and insights on marriage? At what ages is marriage acceptable? Why do you feel this is an acceptable age for marriage?
12. Now, I’d like to ask about your community’s view on marriage. Can you tell us about how this community views marriage? At what age is marriage considered acceptable in this community? Why does the community feel this is an acceptable age for marriage?
13. How do you work with girls and their families to navigate marriage practices? Is this through programs (holistic or focused)? Is it through informal support?
    1. Can you describe a time when you worked with a girl and her family on the issue of marriage?
14. Do you think that issues faced by adolescent girls need to be addressed through adolescent-centered programs (either by the government, NGOs, or other social service providers)?
    1. What do you think needs to be done?

*Impact of COVID-19 on Marriage Practices*

1. Have you recognized a change in the number child marriages since COVID-19? Increase or decrease?
   - 1. If yes, *how* has COVID-19 driven this change (e.g., restricted access to services/education; isolation)?
2. Since COVID-19, have you recognized a change in drivers of CEFM? If so, how have they changed (type, magnitude, key actors etc.)
3. Since COVID-19, have you recognized a change in how decisions within households are being made around availability and access to resources (e.g., finances, food, education, health services, freedom of movement, social connection etc)?

*Closing*

Thank you so much for taking the time to answer these questions. We very much appreciate your time, as your input will help to shape this research effort going forward.

Is there anything else you’d like to add about the topics we discussed?

**Key Informant Interview Guide: Service Providers and Health Workers**

*Background and Context*

First, I’d like to ask you some general questions about your experience working in this setting.

1. Can I start by asking a little about yourself? What is your current role and length of time in this position?
2. Do you have experience working with adolescents and their families in this role or in previous roles?
   1. **If yes**: can you tell me more about your experience?
3. How do you define adolescence? What marks the boundaries of adolescence? In other words, when do girls become women?
4. In your view, what are the most pressing issues facing adolescent girls in this setting?
   1. Are there any differences in issues faced by younger adolescence (10-14 years of age) and older adolescents (those 15-19 years of age)?
      1. **If yes**, can you tell me more about the differences.
   2. What about adolescent girls living with a disability?
5. In your view, what are the most pressing issues facing adolescent girls in this setting?
   1. Are there any differences in issues faced by younger adolescents (10-14 years of age) and older adolescents (those 15-19 years of age)?
      1. **If yes**, can you tell me more about the differences?
   2. What about adolescent girls living with a disability?
6. What kinds of services or support are available for adolescent girls in this setting?
7. Which actors (e.g., CSO, NGOs, INGOs, humanitarian, government) provides these services?
8. What are the barriers faced by girls in accessing these services/support?
9. What service/support gaps exist in this community?
10. Who makes the decision for girls to participate in these services?
11. Does your [organization/clinic/employer] work directly with these influencers?

[If respondents works with adolescents] When implementing adolescent programming, what are the key challenges that you experienced in trying to reach adolescent girls?

*Marriage Practices and Programming*

1. Can you tell us about your views and insights on marriage? At what ages is marriage acceptable? Why do you feel this is an acceptable age for marriage?
2. Now, I’d like to ask about your community’s view on marriage. Can you tell us about how this community views marriage? At what age is marriage considered acceptable in this community? Why does the community feel this is an acceptable age for marriage?
3. How do you work with girls and their families to navigate marriage practices? Is this through programs (holistic or focused)? Is it through informal support?
4. Can you describe a time when you worked with a girl and her family on the issue of marriage?
5. Do you know of any specific programs in this community that address child, early, and forced marriage (CEFM)?
   1. **If yes**:
      1. Are you directly involved in such projects?
      2. Have you partnered with other agencies or organizations on CEFM programming?
      3. What are the key challenges experienced by CEFM programs in trying to reach adolescent girls?
   2. **If no:**
      1. Do you think there is an urgent need for CEFM programming here in your community? Why or Why not?
6. What challenges do you think adolescent girls face in terms of accessing information and services?
   1. Are there any challenges regarding attitudes of service providers? Or program staff?
   2. How have these challenges been addressed?
7. What barriers (at the policy, community, facility, or family level) might be experienced in providing [CEFM; or sexual and reproductive health; GBV; child protection] services to adolescents in this community?
8. To what extent are service providers supporting data collection and reporting on CEFM with gender-sensitive indicators and sex-disaggregated data?
9. Are there CEFM, or adolescent [sexual and reproductive health; GBV; child protection] initiatives underway, in neighboring areas, that have a strong model for service delivery which could be learned from? Can you tell me what made those initiatives successful?

*Impact of COVID-19 on Marriage Practices*

1. What has been the overall impact of COVID-19 on CEFM practices in the communities where you work?
   1. Have you recognized a change in the incidence of CEFM cases since COVID-19? Increase or decrease?
      1. If yes, how has COVID-19 driven this change (e.g., restricted access to services/education; isolation)?
   2. How has COVID-19 altered decision making pathways within households around availability and access to resources defined as human, social, and cultural capital required for healthy child development and wellbeing?

*Impact of COVID-19 on Marriage Practices*

1. What has been the overall impact of COVID-19 on CEFM practices in the communities where you work?
   1. Have you recognized a change in the incidence of CEFM cases since COVID-19? Increase or decrease?
      1. If yes, *how* has COVID-19 driven this change (e.g., restricted access to services/education; isolation)?
2. Have the drivers of CEFM changed? If yes, how have they changed (e.g., type, magnitude, key actors etc.)
   1. What are the impacts of these changes in the present and what are the anticipated impacts?
3. How has COVID-19 altered decision-making pathways within households around availability and access to resources defined as human, social, and cultural capital required for healthy child development and wellbeing (e.g., money; access to services; freedom of movement; social connections; education; food/internet etc.)?

*Coordination*

1. What is your institution’s role and how does it relate to the issue of CEFM?
2. Is there a national strategy or legal framework to address the needs of adolescent girls? What are some of the key national policies/laws that are relevant to the issue of CEFM for the persons affected by food insecurity in this setting?
   1. What about policies/laws relating to:
      1. Protection/GBV
      2. Other harmful traditional practices: FGC, and others
      3. Livelihoods/earnings
      4. Education-access of displaced to primary and secondary school
      5. Health/Education-SRH within health education curriculum
      6. Age of consent for medical services
      7. Other
3. How have different sectors (education-formal and non-formal, social service, protection, recreation and health) engaged in providing services to adolescents in this setting?
   1. Can you share specific interventions or programs underway?
   2. What challenges have been faced by such programs?
   3. Can you tell me the categories of girls that might be excluded from programs and services?
   4. Do you feel any of these programs have improved SRH for adolescents as a result?

*Adolescent Services*

1. Who provides SRH education within this community? How is information shared with adolescent girls about the following topics:
   1. Puberty
   2. Menstruation and menstrual hygiene
   3. Fertility
   4. Communication between men and women
   5. STIs and HIV
   6. Contraception and family planning
   7. Birth spacing
   8. Pregnancy complications and warning signs
   9. Marriage
2. What social service and protection programs are available for adolescent girls in this community? Are there different programs available for younger adolescent girls (12-14) than older adolescent girls (15-19)? For adolescent girls living with a disability? For girls in-school versus out-of-school? For indigenous and other minority populations?
3. What health education services or programs are available for adolescents in this community? Are there more services available for younger adolescents (12-14) than older adolescents (15-19)? For adolescent girls living with a disability? For girls in-school versus out-of-school? For indigenous and other minority populations?

*Recommendations for Adolescent Programming*

1. What information would be helpful to know about the adolescent girls, in order to provide services which meet their needs?
2. How do you think evidence about the drivers of CEFM and protective assets/capacities of adolescent girls can be used to inform services or programs?
   1. Are there other organizations (e.g., advocacy, programming, or research), who might be particularly interested in this research?
   2. What other data would be helpful to gather in order to develop a program to prevent and respond to CEFM in this setting?
3. What recommendations do you have to strengthen/promote actions to prevent or stop girls CEFM?
4. What are the key areas that you think need to be addressed and how?
5. What would the next steps be to accomplish this? What committees/government/community actors would need to be involved and in what ways?

*Closing*

Thank you so much for taking the time to answer these questions. We very much appreciate your time, as your input will help to shape this research effort going forward. Is there anything else you’d like to add about the topics we discussed?

**Key Informant Interview Guide: UN and Government Representatives**

*Background and Context*

First, I’d like to ask you some general questions about your experience working in this setting.

1. Can I start by asking a little about yourself? What is your current role and length of time in this position?
2. Do you have experience working with adolescents and their families in this role or in previous roles?
   1. **If yes**: can you tell me more about your experience?
3. How do you define adolescence? What marks the boundaries of adolescence? In other words, when do girls become women?
4. In your view, what are the most pressing issues facing adolescent girls in this setting?
   1. Are there any differences in issues faced by younger adolescence (10-14 years of age) and older adolescents (those 15-19 years of age)?
      1. **If yes**, can you tell me more about the differences.
   2. What about adolescent girls living with a disability?
5. What kinds of services or support are available for adolescent girls in this setting?
6. Which actors (e.g., CSO, NGOs, INGOs, humanitarian, government) provides these services?
7. What are the barriers faced by girls in accessing these services/support?
8. What service/support gaps exist in this community?
9. Who makes the decision for girls to participate in these services?
10. Does your [organization/government department] work directly with these influencers?
11. When implementing adolescent programming, what are the key challenges that you experienced in trying to reach adolescent girls?

*Marriage Practices & Response*

1. Can you tell us about your views and insights on marriage? At what ages is marriage acceptable? Why do you feel this is an acceptable age for marriage?
2. Now, I’d like to ask about your community’s view on marriage. Can you tell us about how this community views marriage? At what age is marriage considered acceptable in this community? Why does the community feel this is an acceptable age for marriage?
3. How do you work with girls and their families to navigate marriage practices? Is this through programs (holistic or focused)? Is it through informal support?
   1. Can you describe a time when you worked with a girl and her family on the issue of marriage?
4. Do you know of any specific programs here that address child, early, and forced marriage (CEFM)?
   1. **If yes**:
      1. Are you directly involved in such projects?
      2. Have you partnered with other agencies or organizations on CEFM programming?
      3. What are the key challenges experienced by CEFM programs in trying to reach adolescent girls?
   2. **If no**:
      1. Do you think there is an urgent need for CEFM programming here in your community? Why or Why not?
5. What challenges do you think adolescent girls face in terms of receiving CEFM information and services?
   1. Are there any challenges regarding attitudes of service providers? Or program staff?
   2. How have these challenges been addressed?
6. What barriers (at the policy, community, facility, or family level) might be experienced in providing [CEFM; or sexual and reproductive health; GBV; child protection] services to adolescents in this community?
7. Are there CEFM, or adolescent [sexual and reproductive health; GBV; child protection] initiatives underway, in neighboring areas, that have a strong model for service delivery which could be learned from? Can you tell me what made those initiatives successful?
8. From your perspective as a (choose appropriate) government official, ministry official, donor, or UN contact, what priorities exist for providing services to the adolescent girls affected by food insecurity in Chiredze?

*Impact of COVID-19 on Marriage Practices*

1. What has been the overall impact of COVID-19 on CEFM practices in the communities where you work?
   1. Have you recognized a change in the incidence of CEFM cases since COVID-19? Increase or decrease?
      1. If yes, *how* has COVID-19 driven this change (e.g., restricted access to services/education; isolation)?
2. Have the drivers of CEFM changed? If so, how have they changed (type, magnitude, key actors etc.)
   1. What are the impacts of these changes in the present and what are the anticipated impacts?
3. How has COVID-19 altered decision-making pathways within households around availability and access to resources defined as human, social, and cultural capital required for healthy child development and wellbeing (e.g., money, food, access to education/health services/programming; freedom of movement; social connection, etc.)?

*Coordination*

1. Are there specific priorities within each sector: health, protection, GBV, education, and livelihoods?
2. What is the process for determining these priorities? Does the national strategy on adolescent girls inform these priorities?
3. What CEFM/protection services are currently funded for adolescent girls in this setting?
4. What type of variations in support do you see for different sub-populations: by age group (15-19 years and 14 years and younger), marital status, gender, schooling status, or disability?
5. What opportunities might exist across these sectors to address CEFM for adolescent girls?
6. What challenges might occur in addressing CEFM for adolescent girls?
7. To what extent is there coordination across sectors working with adolescents?
8. Which donors have engaged in these programs for adolescents?

*Recommendations for Adolescent Programming*

1. What information would be helpful to know about the adolescent girls, in order to provide services which meet their needs?
2. How do you think evidence about the drivers of CEFM and protective assets/capacities of adolescent girls can be used to inform services or programs?
   1. Are there other organizations (e.g., advocacy, programming, or research), who might be particularly interested in this research?
   2. What other data would be helpful to gather in order to develop a program to prevent and respond to CEFM in this setting?
3. What recommendations do you have to strengthen/promote actions to prevent or stop girls CEFM?
4. What are the key areas that you think need to be addressed and how?
5. What would the next steps be to accomplish this? What committees/government/community actors would need to be involved and in what ways?

*Closing*

Thank you so much for taking the time to answer these questions. We very much appreciate your time, as your input will help to shape this research effort going forward. Is there anything else you’d like to add about the topics we discussed?
